# Lipidomic and metabolic changes in the P4-type ATPase ATP10D deficient C57BL/6J wild type mice upon rescue of ATP10D function

**DOI:** 10.1371/journal.pone.0178368

**Published:** 2017-05-25

**Authors:** Alexander Sigruener, Christian Wolfrum, Alfred Boettcher, Thomas Kopf, Gerhard Liebisch, Evelyn Orsó, Gerd Schmitz

**Affiliations:** 1 Institute of Clinical Chemistry and Laboratory Medicine, Regensburg University Medical Center, Regensburg, Germany; 2 Institute of Molecular Systems Biology, ETH Zürich, Zürich, Switzerland; University of Melbourne, AUSTRALIA

## Abstract

**Background:**

Sequence variants near the human gene for P4-type ATPase, class V, type 10D (*ATP10D*) were shown to significantly associate with circulating hexosylceramide d18:1/16:0 and d18:1/24:1 levels, obesity, insulin resistance, plasma high density lipoprotein (HDL), coronary stenotic index and intracranial atherosclerotic index. In mice *Atp10d* is associated with HDL modulation and C57BL/6 mice expressing a truncated, non-functional form of ATP10D easily develop obesity and insulin resistance on high-fat diet.

**Results:**

We analyzed metabolic differences of ATP10D deficient C57BL/6J wild type and ATP10D transgenic C57BL/6J BAC129 mice. ATP10D transgenic mice gain 25% less weight on high-fat diet concomitant with a reduced increase in fat cell mass but independent of adipocyte size change. ATP10D transgenic mice also had 26% lower triacylglycerol levels with approximately 76% bound to very low density lipoprotein while in ATP10D deficient wild type mice 57% are bound to low density lipoprotein. Furthermore increased oxygen consumption and CO_2_ production, 38% lower glucose and 69% lower insulin levels and better insulin sensitivity were observed in ATP10D transgenic mice. Besides decreased hexosylceramide species levels were detected. Part of these effects may be due to reduced hepatic stearoyl-CoA desaturase 1 (SCD1) expression in ATP10D transgenic mice, which was reflected by altered fatty acid and lipid species patterns. There was a significant decrease in the hepatic 18:1 to 18:0 free fatty acid ratio in transgenic mice. The ratio of 16:1 to 16:0 was not significantly different. Interestingly both ratios were significantly reduced in plasma total fatty acids.

**Summary:**

In summary we found that ATP10D reduces high-fat diet induced obesity and improves insulin sensitivity. ATP10D transgenic mice showed altered hepatic expression of lipid-metabolism associated genes, including *Scd1*, along with changes in hepatic and plasma lipid species and plasma lipoprotein pattern.

## Introduction

Insulin resistance (IR) is a major constituent of the metabolic syndrome predisposing to type 2 diabetes (T2D) mellitus and cardiovascular complications which both still cause approximately 50% of all deaths in Europe [1]. Even though IR is well studied, the factors leading to the progression of this disease have only been poorly defined. In recent years it has become evident that certain lipid molecular species can cause IR. Especially glucosylceramides have been reported to be involved in the progression of IR and T2D [2]. In the EUROSPAN study, a human genome-wide association between clinical and laboratory phenotypes and single-nucleotide polymorphisms (SNPs), we successfully identified SNPs in serine palmitoyltransferase, long chain base subunit 3, ceramide synthase 4, sphingosine-1-phosphate phosphatase 1, the fatty acid desaturases 1–3 and ATPase, class V, type 10D (*ATP10D*) that associated with plasma levels of certain sphingolipid species. SNPs in the region of the P4 ATPase family gene *ATP10D* showed significant associations with circulating hexosylceramide (HexCer) d18:1/16:0 and d18:1/24:1 (mainly glucosylceramide [3]) and myocardial infarction [4]. Recently, one of these SNPs, rs2351791, was also shown to be associated with plasma high density lipoprotein (HDL), coronary stenotic index and intracranial atherosclerotic index [5].

The P4 ATPase family consists of multispan transmembrane translocases involved in phospholipid transfer from the exoplasmic/luminal side to the cytoplasmic membrane leaflet [6]. The importance of P4 ATPases for maintenance of membrane asymmetry is underlined by ATPase, aminophospholipid transporter, class I, type 8B, member 1 (ATP8B1). Mutations in the human *ATP8B1* gene lead to Progressive Familial Cholestasis type 1 and Benign Recurrent Intrahepatic Cholestasis type 1 [7]. Subcellular localization of P4 ATPases depends on interaction with members of the CDC50 family, representing accessory β-subunits for membrane translocation that may also determine substrate specificity [8]. Recently, it was shown that the P4 ATPase ATP10D in a transmembrane protein 30A (TMEM30A/CDC50A) dependent manner translocates from the endoplasmatic reticulum to the plasma membrane [9].

In mice *Atp10d* is located in a genomic region associated to HDL modulation [10,11]. Interestingly, C57BL/6 mice that express a truncated, non-functional ATP10D protein due to a nonsense mutation [12] are one of the most susceptible strains to develop IR and T2D when exposed to a high-fat diet (HFD) [13]. In this study we analyzed the role of ATP10D in the progression of IR and metabolic homeostasis through the generation of a novel transgenic mouse model in which we rescued the aberrant *Atp10d* expression.

## Material and methods

### Generation of C57BL/6J transgenic BAC129 *Atp10d* mice

Heterozygous C57BL/6J transgenic BAC129 *Atp10d* mice (TG) were generated by pronuclear injection of a bacterial artificial chromosome BAC containing the Sv129 full length *Atp10d* (BAC number BmQ383-E5). Integration of BAC and copy number were determined by qPCR. All animal experiments were approved by the Kantonale Tierversuchskommission des Kantons Zürich (Permit number 135).

All animal were maintained in C57BL/6J background and maintained on a 12 hours light/dark cycle in a pathogen-free animal facility. Groups of animals were fed a high fat diet (Kliba) containing 60% fat for the indicated times. After 8 weeks and fasting overnight loss of consciousness was produced by rising concentrations of CO_2_. Mice were then exsanguinated by cardiac puncture and heart perfused with 0.9% NaCl before liver, visceral adipose tissue, subcutaneous adipose tissue, brown adipose tissue, spleen and heart were collected.

### Metabolic cage analysis

Measurements of food and water intake and O2 consumption / CO2 production is performed non-invasively using an automatic feeding monitoring system coupled to an open-circuit indirect calorimetry system (TSE Phenomaster System). In addition, via infrared light-beam frames detailed measurements of spontaneous home cage activity can be obtained. Mice are single housed in regular type III cages; food and water are available ad libitum and intake can be constantly monitored. Food intake was not altered between the two lines (data not shown). Each cage is connected to the fresh air supply as well as the sample switch unit for drawing air samples from each cage. Cages (n = 12) are enclosed in a ventilated cabinet (TSE Systems) to precisely control ambient temperature and light intensity.

### Insulin tolerance test

The intraperitoneal insulin tolerance test (ITT) measures glucose levels subsequent to a standardized insulin load. It gives an estimate of the insulin sensitivity (IS) of the animals. Animals are fasted for 14 to 16 hrs, afterwards a bolus of insulin is administered intraperitoneally (IP, 1U/kg BW). Blood is withdrawn by tail vein incision at different time points, and glucose is measured.

### Genomic DNA extraction and *Atp10d* Exon 12 amplification

DNA was isolated from 20 mg of liver using the QIAamp DNA Mini Kit (Qiagen). Quantity und purity were measured with Nanodrop (PeqLab). *Atp10d* exon 12 was amplified using the following oligonucleotides: FWD: TCA CGT GTA GCG TCG TTT TC, REV: CTG ACC ATC ACC ATG GCA AC.

### Lipoprotein separation by Fast Phase Liquid Chromatography

Plasma lipoprotein fractions were isolated by Fast Phase Liquid Chromatography as previously published [14]. Cholesterol and triglycerides in the fractions were determined with commercial kits (Roche) on the Cobas Integra 400 plus.

### Native gradient gel electrophoresis

6 μl of plasma were stained with 2 μl (0.1 mg/ml in dimethyl sulfoxide) lipophilic dialkylaminostyryl fluorophore (Di_10_-ASP-PS, Molecular Probes) and diluted with 8 μl 2x native sample buffer (Invitrogen). 5 μl of the sample were separated by 4 to 16% polyacrylamide gradient gel electrophoresis in native running buffer (Invitrogen) at 60 V for 18 h at 10°C. In addition, five reference proteins (HMW-Native Marker Kit, GE-Healthcare) were run simultaneously for calibrating particle sizes: thyroglobulin (17 nm), apoferritin (12.2 nm), catalase (9.2 nm), lactatedehydrogenase (8.2 nm), and BSA (7.1 nm). LDL particles were determined by comparing migration distances to those of proteins of known size [15]. PAGE-gels were scanned on a Typhoon 9400 scanner (GE Healthcare) with an excitation of 488 nm and emission of 610 nm to detect the fluorescence dye bound to the serum lipoproteins.

### RNA extraction

10 mg frozen liver were thawed in RLT buffer containing beta-mercaptoethanol and homogenized using a Precelly 24 (PeqLab; 5000 rpm, 20 sec). After centrifugation (2000 rpm, 10 min) RNA was isolated using the RNeasy tissue mini kit and DNA digestion was performed on column (Qiagen). RNA quality was assessed with Bioanalyzer 2100 (Agilent). Quantity und purity were measured with Nanodrop (PeqLab).

### Micro array and pathway analysis

200 ng of total RNA were processed with the One-Color Quick Amp Labeling Kit according to the manufacturer’s instructions (Agilent). cRNA quantity and labeling efficiency was checked with Nanodrop (PeqLab). Scanning of arrays was performed with the G2565CA (Agilent) (5μM, single pass, 20 bit, no XDR). Micro array scan data were extracted with Feature Extraction software 10.7.3.1 (Agilent). After elimination of noise and flagged signals data were analyzed with ChipInspector (Genomatix). Gene regulated more than 2 fold with a mean signal bigger 100 in at least one of the groups were considered for further analysis.

### TaqMan PCR

TaqMan PCR assays were performed on the ABI Prism 7900 HT Sequence Detection System (Life Technologies) using commercial assays (Life Technologies: 18s rRNA–Hs99999901_s1, Agpat9—Mm04211965_m1, Atp10d - Mm00462762_m1, Cidec—Mm00617672_m1, Fgf21—Mm00840165_g1, Lpin1—Mm00550511_m1, Scd1—Mm01197142_m1, Srebf1—Mm00550338_m1). Relative quantification was carried out as described earlier [16].

### *Atp10d* Exon 12 qPCR

10 ng of liver cDNA were amplified using the following oligonucleotides: FWD: GGC TTC CAA CCT GTG CTA TG, REV: TGA CCA CGA CCG ACA TTC TT.

### *Atp10d* Exon 12 Sanger sequencing

The relevant position was directly sequenced on both strands by the Sanger method (genomic: FWD: GTG CTA TGA GGC GGA GAG AGT C, REV: CTG ACC ATC ACC ATG GCA AC; RNA: amplification oligonucleotides). The sequencing reaction was performed with the BigDye Terminator v1.1 Cycle Sequencing Kit (Life Technologies), purified with the DyeEx 2.0 Spin Kit (Qiagen) and analyzed on a ABI Prism 3130xl (Life Technologies) capillary sequencer.

### Adipocyte size quantification

Adipocyte size was quantified using hematoxylin and eosin stained sections coupled to an automated image analysis using the Cell Profiler Pipeline as described previously [17].

### Protein isolation and immunoblot

Liver tissues (20–30 mg) were homogenized in RIPA buffer (150 mM NaCL, 1% NP-40, 0.5% sodium deoxycholate, 0.1% SDS, 50 mM Tris, pH 8.0) supplemented with protease inhibitors. The extracts were incubated on ice for 20 minutes and then centrifuged at 15,000 g 15 min to remove tissue debris. Protein concentrations were determined by bicinchoninic acid assay (Pierce) and 15 μg of lysates were subjected to SDS-polyacrylamide gel electrophoresis on 4–12% Bis-Tris polyacrylamide gels with MOPS running buffer (Invitrogen). Proteins were transferred onto polyvinylidene difluoride-membranes (BioRad) which were then blocked by incubation for 1 h with 5% nonfat dry milk in PBS. Primary antibodies (anti-SCD1 (C12H5) Rabbit-mAb (Cell Signaling: 2794), anti-FGF21 (EPR8314) Rabbit-mAb (abcam: ab171941) or anti-ß-actin (AC-74) mouse monoclonal (Sigma: A5316)) were diluted in 1% nonfat dry milk in PBS with 0.1% Tween 20 and interacting anti-rabbit-or anti-mouse-horseradish peroxidase-IgG (Jackson ImmunoResearch) was detected by chemiluminescence ECL-Pus blot detection system (GE-Healthcare). Quantification was done with ImageJ software [18].

### Lipid analysis

1 ml of a 1:1 mixture of water and methanol was added to 50 mg tissue. The samples were homogenized using a Precelly 24 (PeqLab; 2 x 20 sec 6500rpm, ice 30 sec, 20 sec 6500 rpm).

Hepatic triacylglycerols (TAG) were extracted according Bligh and Dyer [19] and determined by thin-layer-chromatography. Samples were dried in a SpeedVac and dissolved in 100 μl of 1:1 chloroform / methanol. A high performance thin layer chromatography silica gel 60 (Merck) plate was conditioned for 30 min at 150°C before applying the samples. The plate was subsequently developed with three solvent mixtures: First 1:1 ethyl acetate / heptane, followed by 50:47.5:2.5 diethyl ether / hexane / acetic acid and finally 3:97 diethyl ether / hexane. Staining was done with copper(II) sulfate and heating to 150°C for 30 min. Afterwards the plate was scanned at 346 nm.

Lipid species were quantified by direct flow injection electrospray ionization tandem mass spectrometry using the analytical setup and strategy as previously published [20–22]. A precursor ion scan of *m/z* 184 Da specific for phosphocholine containing lipids was used for phosphatidylcholine (PC), lyso-PC (LPC) and sphingomyelin (SM) [21,22]. Ceramide was analyzed using a fragment ion of *m/z* 264 Da [20]. Neutral loss fragments were used for the following lipid classes: Phosphatidylethanolamine (PE) 141 Da, phosphatidylserine 185 Da, phosphatidylglycerol (PG) 189 Da and phosphatidylinositol 277 Da [23]. PE-based plasmalogens were analyzed according to the principles described by Zemski-Berry [24]. Free cholesterol and cholesteryl ester (CE) were quantified using a fragment ion of *m/z* 369 Da after selective derivatization of free cholesterol [25]. For each lipid class two non-naturally occurring internal standards were added and quantification was achieved by calibration lines generated by addition of naturally occurring lipid species to plasma. Liquid chromatography coupled to tandem mass spectrometry was used to quantify HexCer, lactosylceramides, sphingoid bases, sphingosylphosphorylcholine [26], lysophospholipids sphingosine-1-phosphate and lysophosphatidic acid [27] as well as cardiolipin, bis(monoacylglycero)phosphate, PG and phosphatidic acid [28].

Deisotoping and data analysis for all lipid classes was performed by self programmedself-programmed Excel Macros according to the principles described previously [22]. Lipid species were annotated according to the “Shorthand Notation for Lipid Structures Derived from Mass Spectrometry” [29]. Glycerophospholipid species were annotated based on assumption of even numbered carbon chains only. SM species annotation is based on the assumption that d18:1 (dihydroxy 18:1 sphingosine) is the main base.

Total and non-esterified fatty acids were determined by gas chromatography/mass spectrometry as previously described [30,31].

### Statistical methods

Statistical analyses were performed using the Student´s t-test.

### Principal component analysis (PCA)

PCA analysis was performed using the Excel add-in Multibase package (Numerical Dynamics, Japan).

## Results

### Generation of *Atp10d* transgenic animals

To get more insight into the role of ATP10D a transgenic mouse model was created on the background of the commonly used C57BL/6J laboratory strain that was previously shown to express no functional ATP10D [12]. Sequencing of exon 12 of *Atp10d* confirmed presence of the functional C-allele (NM_153389.3; c.2448) in hepatic genomic DNA and RNA of *Atp10d* transgenic C57BL/6J mice (S1 Fig).

### Metabolic effects of ATP10D in HFD

**Fig 1:**
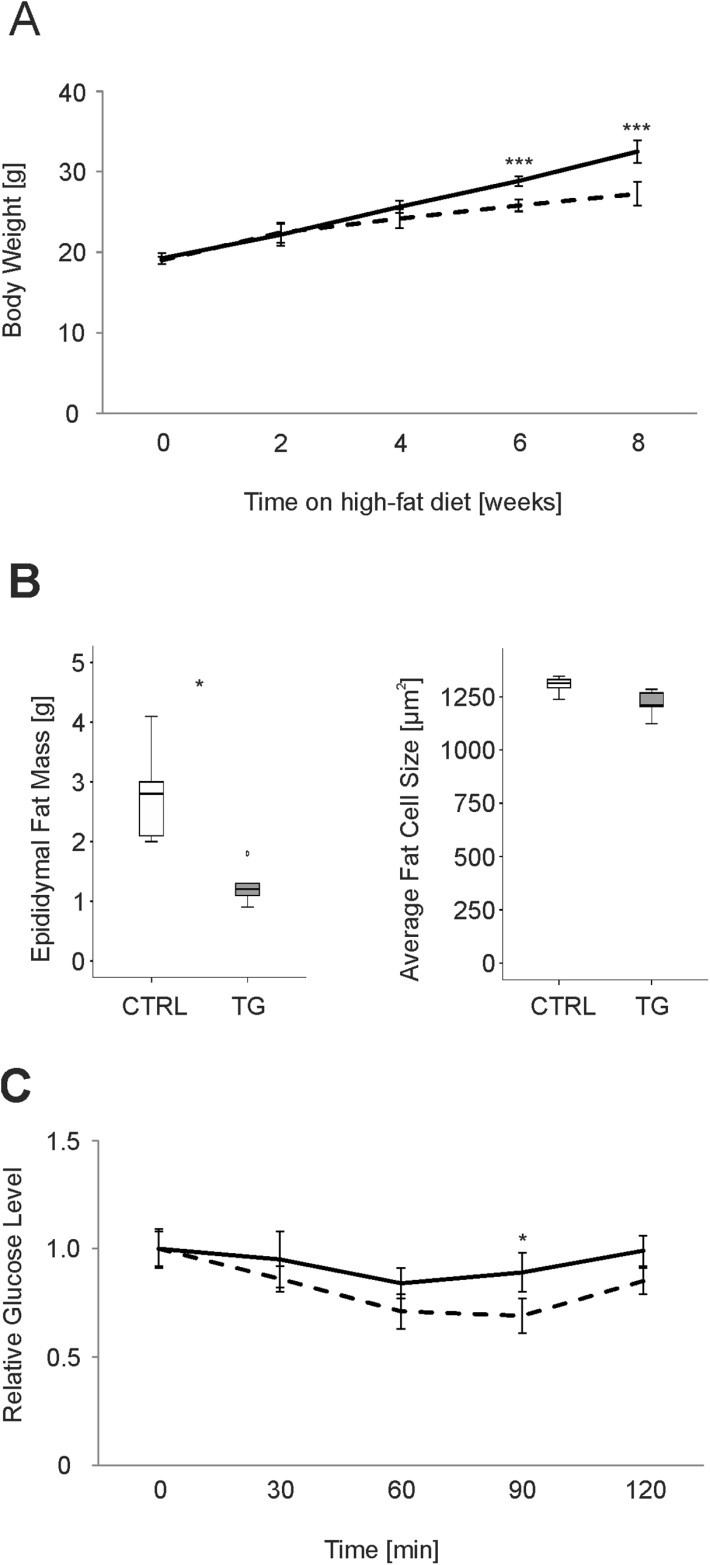
Metabolic differences in ATP10D deficient and transgenic mice on high fat diet. A. Body weight gain over eight weeks on high fat diet. B. Epididymal fat mass and adipocyte size after eight weeks on high fat diet. C. Insulin tolerance of ATP10D deficient and transgenic mice after eight weeks on high fat diet. ATP10D deficient, solid line; ATP10D transgenic, dashed line. CTRL, ATP10D deficient; TG, ATP10D transgenic; *p<0.05; ***p<0.001.

At baseline 6 weeks old male CTRL-mice and TG-mice had similar body weight (CTRL: 19.2±0.7g; TG: 19.0±0.5g). After 8 weeks on HFD the transgenic mice showed a significantly lower weight gain (44%) than the CTRL-mice (69%) (Fig 1A), concomitant with a lesser increase in epididymal fat cell mass. Average fat cell size remained unchanged (Fig 1B). No differences in weight gain and body weight were observed between the two lines when fed a standard chow diet (data not shown).

Since we observed a significant difference in body and fat weight between both groups after 8 weeks on HFD, metabolic parameters and plasma lipoprotein fractions were analyzed in more detail. In the TG-mice plasma glucose (38%) and insulin (69%) were significantly decreased compared to the CTRL-mice. In agreement with these data TG-mice also revealed better IS (Fig 1C). While TAG levels in plasma were significantly decreased by 26% compared to the CTRL-mice, free fatty acids (FFA) and cholesterol remained unchanged in TG-mice (Table 1). Interestingly, TG-mice exhibited reduced hepatic FFA and elevated TAG levels (Table 1), resulting in a significantly (p<0.05) decreased FFA to TAG ratio in liver (CTRL: 0.033±0.006; TG: 0.017±0.003) indicating improved TAG storage. Furthermore, hepatic and plasma HexCer levels were approximately twofold decreased in TG-mice. O_2_-consumption and CO_2_-production were both increased by 10% in TG-mice (Table 1). No significant differences in the respiratory exchange ratio and movement were observed between the two groups.

**Table 1:** Metabolic differences between ATP10D deficient and transgenic mice on high fat diet.

	**CTRL**	**SD**	**TG**	**SD**	**p value**
**Metabolic Parameters—Plasma**					
Glucose [mmol/L]	***7*.*9***	0.9	***4*.*9***	1.1	0.031
Insulin [ng/mL]	***3*.*6***	0.5	***1*.*1***	0.5	0.012
TAG [mg/dL]	***128*.*4***	13.4	***94*.*6***	8.7	0.042
FFA [mmol/L]	1.0	0.1	1.0	0.2	0.084
Cholesterol [mg/dL]	94.6	8.5	83.9	7.9	0.085
**Hepatic TAG and FFA [ng/μg tissue]**					
	**CTRL**	**SD**	**TG**	**SD**	**p value**
FFA	***5*.*6***	0.3	***4*.*4***	0.3	0.001
TAG	***174*.*7***	37.6	***255*.*8***	48.0	0.019
**Hepatic and plasma d18:1/16:0 + d18:1/24:1 hexosylceramide**					
	**CTRL**	**SD**	**TG**	**SD**	**p value**
Plasma [μmol/L]	***0*.*77***	0.15	***0*.*34***	0.04	0.002
Liver [pmol/mg]	***8*.*20***	0.94	***4*.*08***	0.64	0.00008
**Metabolic Parameters—Others**					
	**CTRL**	**SD**	**TG**	**SD**	**p value**
VO_2_ [L/kg/h]	***4537***	189	***5028***	165	0.024
VCO_2_ [L/kg/h]	***3164***	102	***3522***	89	0.021
RER	0.9	0.1	0.9	0.1	0.523
Movement [AU]	4855	946	4912	938	0.834

Significant differences between both groups are shown in bold and italics. CTRL, ATP10D deficient; SD, standard deviation; TG, ATP10D transgenic; TAG, triacylglycerols; FFA, free fatty acids; VO_2_, O_2_ consumption; VCO_2_, CO_2_ production; RER, respiratory exchange ratio, AU, arbitrary units. n = 5.

**Fig 2:**
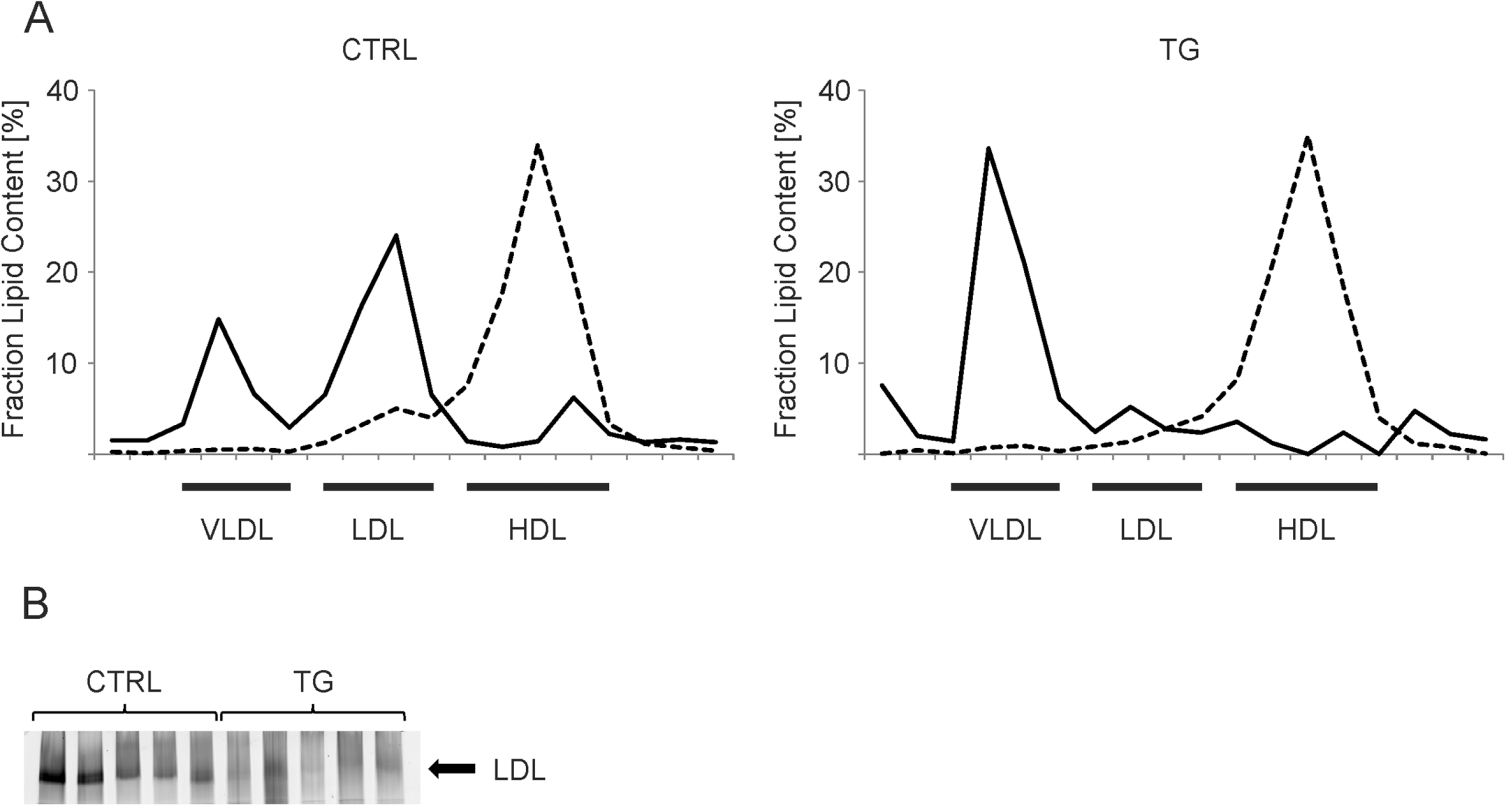
Analysis of plasma lipoprotein fractions of ATP10D deficient and transgenic mice on high fat diet. A. Percentage of total triacylglycerol (solid line) and cholesterol per fraction (dashed line) of Fast Phase Liquid Chromatography isolated plasma lipoprotein in plasma pools of CTRL- and TG-mice. B. Native gradient gel electrophoresis of the individual plasma samples (n = 5). The LDL region was calculated by comparison with the migration distances of proteins of known size [15] and serum LDL was detected using a lipophilic fluorescence dye. CTRL, ATP10D deficient; TG, ATP10D transgenic; VLDL, Very Low Density Lipoprotein; LDL, Low Density Lipoprotein; HDL, High Density Lipoprotein.

In TG-mice the vast majority (75.8%) of lipoprotein-bound TAG was found in the very low density lipoprotein (VLDL) fractions and only 15.5% localized in the low density lipoprotein (LDL) fractions (Fig 2A). In CTRL-mice however, only 30% of TAG were found in the VLDL fraction, while TAG content of the LDL fractions was at approximately 57% (Fig 2A). The TG-mice showed a reduced cholesterol content in the LDL fractions compared to CTRL-mice, indicating either less or cholesterol poorer LDL particles in the TG-mice (Fig 2A). We confirmed these changes in LDL levels by native gradient gel electrophoresis with the individual plasma samples (Fig 2B). Taken together we demonstrate that re-expression of a functional *Atp10d* allele in C57BL/6J mice results in reduced susceptibility to weight gain and the development of IR. In addition re-expression of a functional allele leads to changes in LDL content and composition.

### Lipid profiling of different tissues

Free cholesterol and individual species of SM, PC, PC ethers, PE, PE-based plasmalogens, phosphatidylserine, phosphatidylinositol, LPC, ceramide, HexCer, lactosylceramide, lysophosphatidic acid, bis(monoacylglycero)phosphate, cardiolipin, lyso-PG, phosphatidic acid, PG and CE were measured in liver, visceral adipose tissue, subcutaneous adipose tissue, brown adipose tissue, spleen and heart (S1 Data).

**Fig 3:**
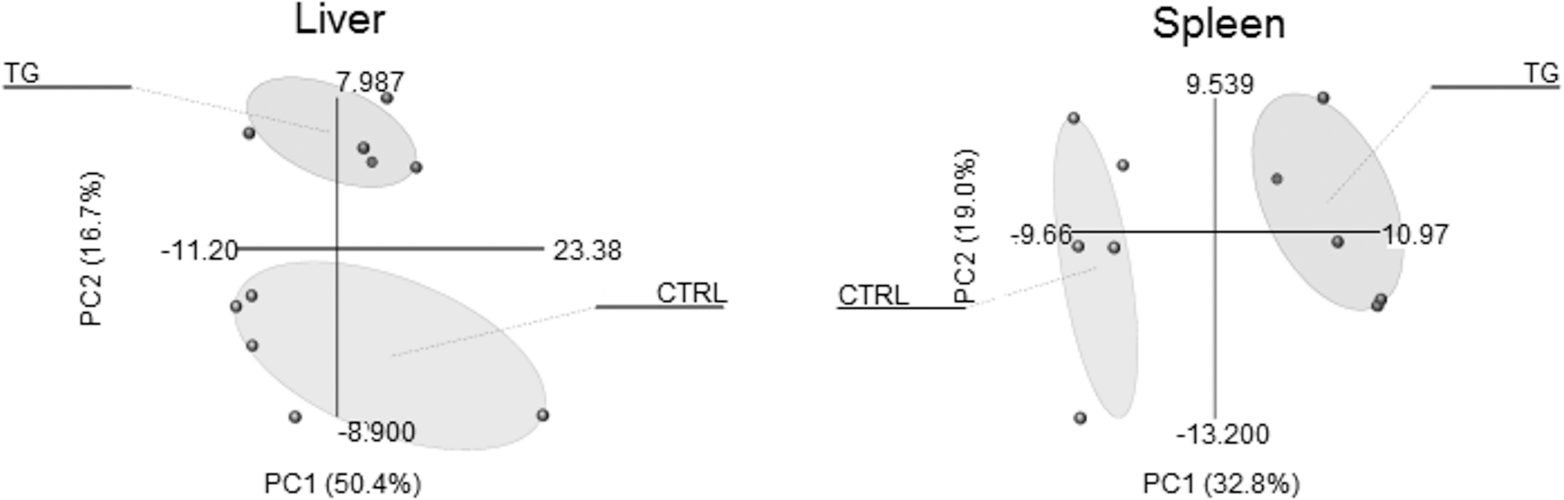
Principal component analysis of the lipid profiles of ATP10D deficient and transgenic mice on high fat diet. A. PCA of the hepatic lipid profiles. Goodness of fit and prediction ability were 67% and 36%. B. PCA of the lipid profiles of spleen. Goodness of fit and prediction ability were 52% and 38%. Distances between single mice are shown (for details see S1 Table).

PCA revealed no clear discrimination of CTRL-mice from the transgenic mice when comparing the lipid profiles of visceral adipose tissue, subcutaneous adipose tissue, brown adipose tissue and heart. In contrast CTRL-mice and transgenic mice were clearly separated by their hepatic lipid profiles. Besides the liver also in spleen re-expression of ATP10D leads to lipid profile changes that allow clearly separation of CTRL-mice and transgenic mice (Fig 3). The contribution of each lipid species is shown in S1 Table.

### Transcriptional effects of ATP10D in HFD

**Fig 4:**
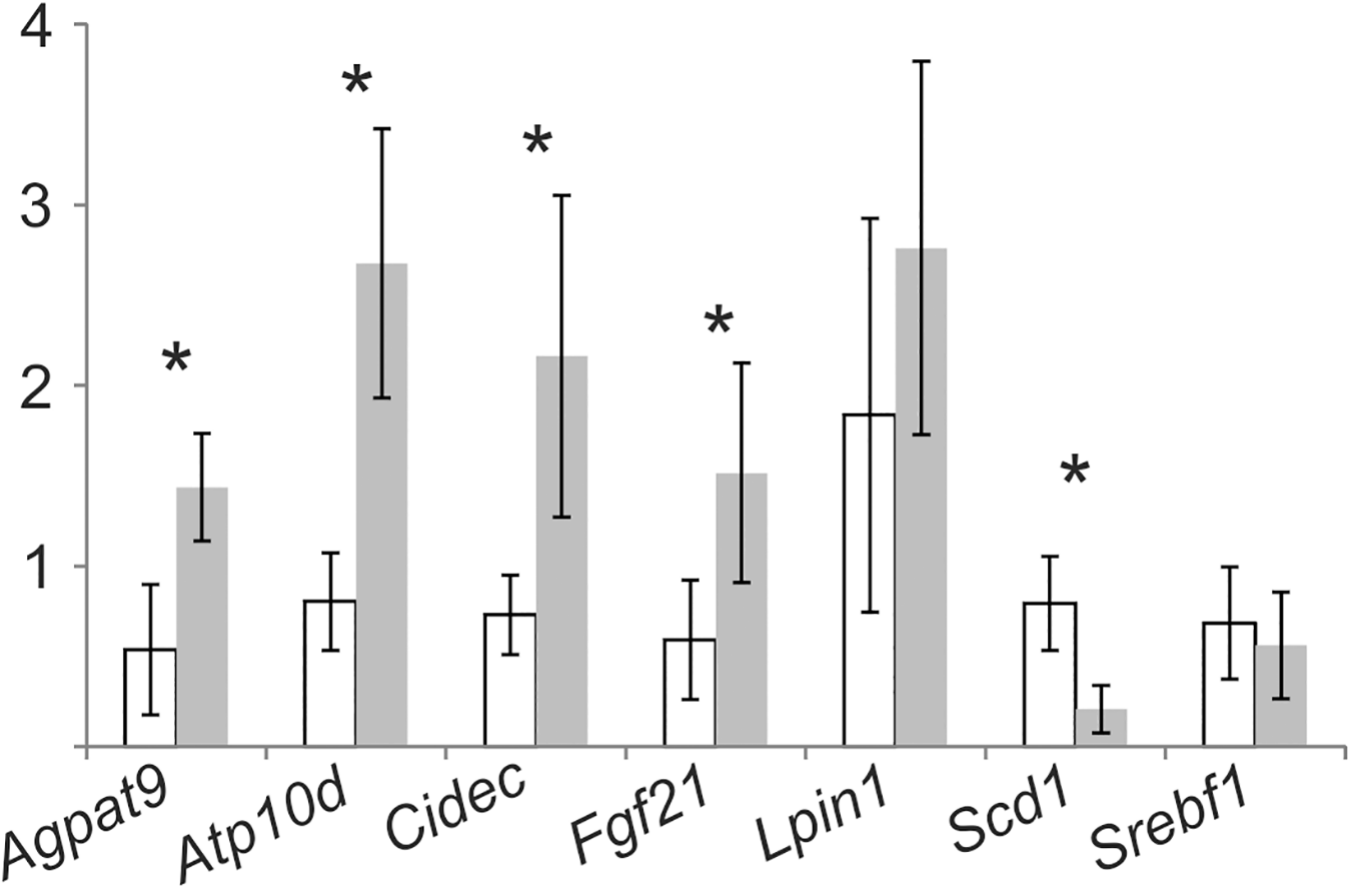
Expression of selected genes in ATP10D deficient and transgenic mice on high fat diet. Expression was measured by TaqMan real-time PCR and normalized to 18S rRNA. Mean values of CTRL- and TG-mice are shown relative to CTRL-mouse1 together with the standard deviation (n = 5). ATP10D deficient, white bars; ATP10D transgenic, grey bars. *p<0.05.

Based on the observed alteration in the hepatic lipid profiles and given the key role of the liver in lipoprotein metabolism we analyzed hepatic mRNA expression of CTRL- and TG-mice in more detail by Agilent microarrays. 114 genes with significantly different expression were detected and classified according to MeSH (S2 Table). Among the top ten MeSH-Terms found were obesity, lipodystrophy, overweight and overnutrition (Table 2) comprising 28 genes (S2 Table). Cell death-inducing DFFA-like effector c (*Cidec*), fibroblast growth factor 21 (*Fgf21*), phosphatidic acid phosphohydrolase lipin 1 (*Lpin1*), pituitary tumor-transforming 1, renin 1 structural, delta-9-desaturase stearoyl-Coenzyme A desaturase 1 (*Scd1*) and sterol regulatory element binding transcription factor 1 (*Srebf1*) were found in all categories. The significantly altered expression of 1-acylglycerol-3-phosphate O-acyltransferase 9 (*Agpat9*), *Cidec*, *Fgf21*, *Scd1* and *Atp10d* was confirmed by TaqMan RT PCR (Fig 4). In accordance with the array data the expression of *Lpin1* was not significantly different in TG-mice (Fig 4). Changed expression of *Srebf1* could not be confirmed (Fig 4). Given the key role of *Scd1* in fatty acid metabolism we focused further analysis on *Scd1*.

**Table 2:** MeSH classification of differentially regulated genes between ATP10D deficient and transgenic mice on high fat diet.

MeSH-Term	MeSH-Term id(s)	p value	Genes observed	Genes expected
Liver Diseases	• C06.552	0.00003	45	27
***Obesity***	• C23.888.144.699.500	0.00009	27	13
***Lipodystrophy***	• C18.452.880.391 • C17.800.849.391 • C18.452.584.625	0.00010	8	1
***Overweight***	• C23.888.144.699	0.00011	27	13
***Obesity***	• C18.654.726.500	0.00017	27	13
***Overnutrition***	• C18.654.726	0.00017	27	13
Connective Tissue	• C17.300	0.00024	32	18
Liver Neoplasms	• C06.552.697 • C06.301.623 • C04.588.274.623	0.00036	36	21
Skin Diseases, Metabolic	• C18.452.880 • C17.800.849	0.00047	9	2
Atherosclerosis	• C14.907.137.126.307	0.00055	18	8

Obesity associated categories are shown in bold and italics.

### ATP10D alters Scd1 expression and function under HFD

**Fig 5:**
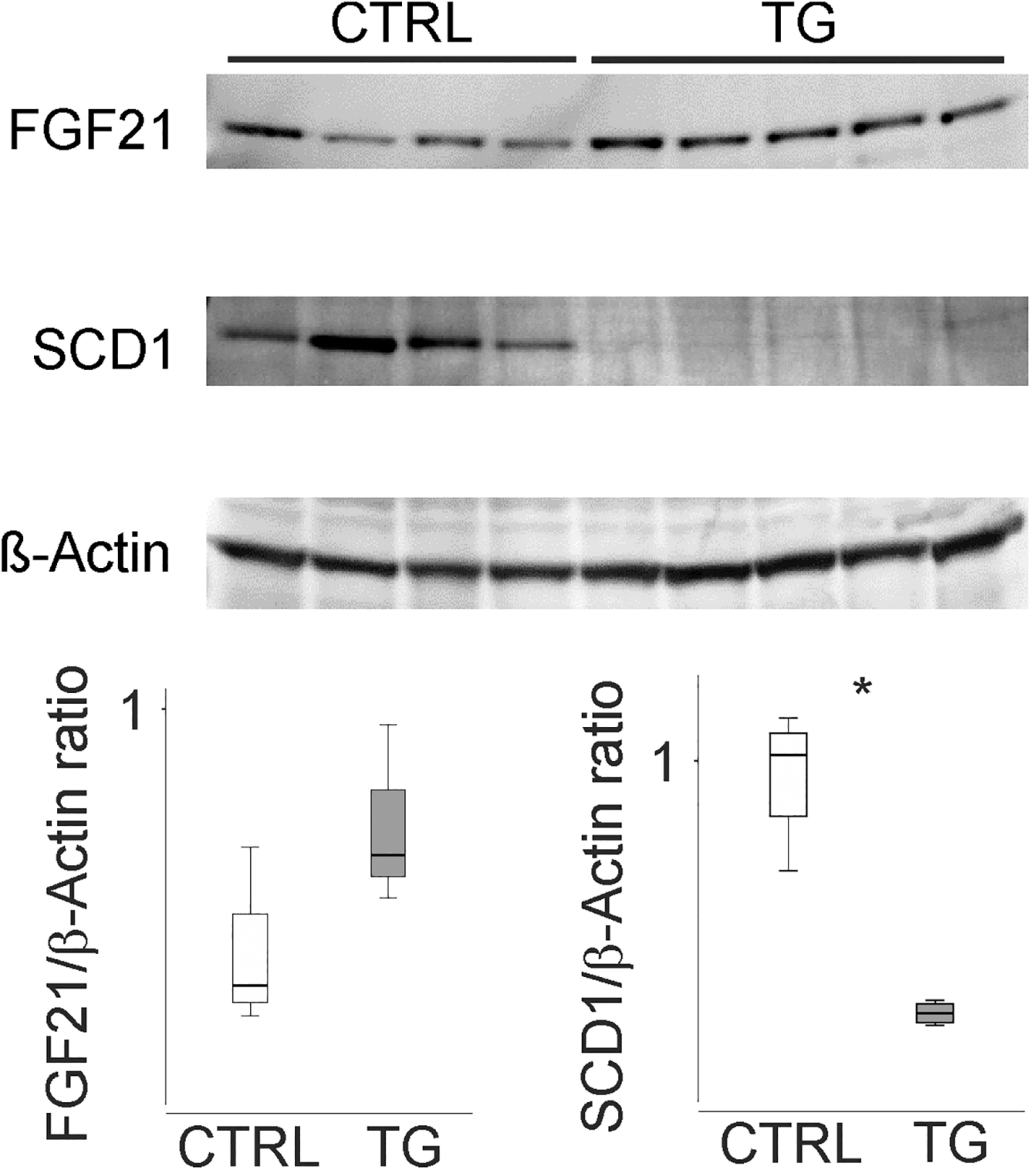
Hepatic protein levels of FGF21 and SCD1 of ATP10D deficient and transgenic mice on high fat diet. Immunoblot of FGF21, SCD1 and beta-actin in the individual liver samples. Data were quantified using ImageJ software [18] and are shown relative to β-Actin as housekeeping protein. CTRL, ATP10D deficient; TG, ATP10D transgenic; *p<0.05.

Gene array and TaqMan analysis revealed decreased levels of *Scd1* in liver of TG-mice (S2 Table and Fig 4). Decreased expression of SCD1 in liver of TG-mice could also be confirmed at the protein level (Fig 5). In agreement with mRNA data hepatic FGF21 protein expression appears slightly higher in TG-mice (Fig 5).

**Fig 6:**
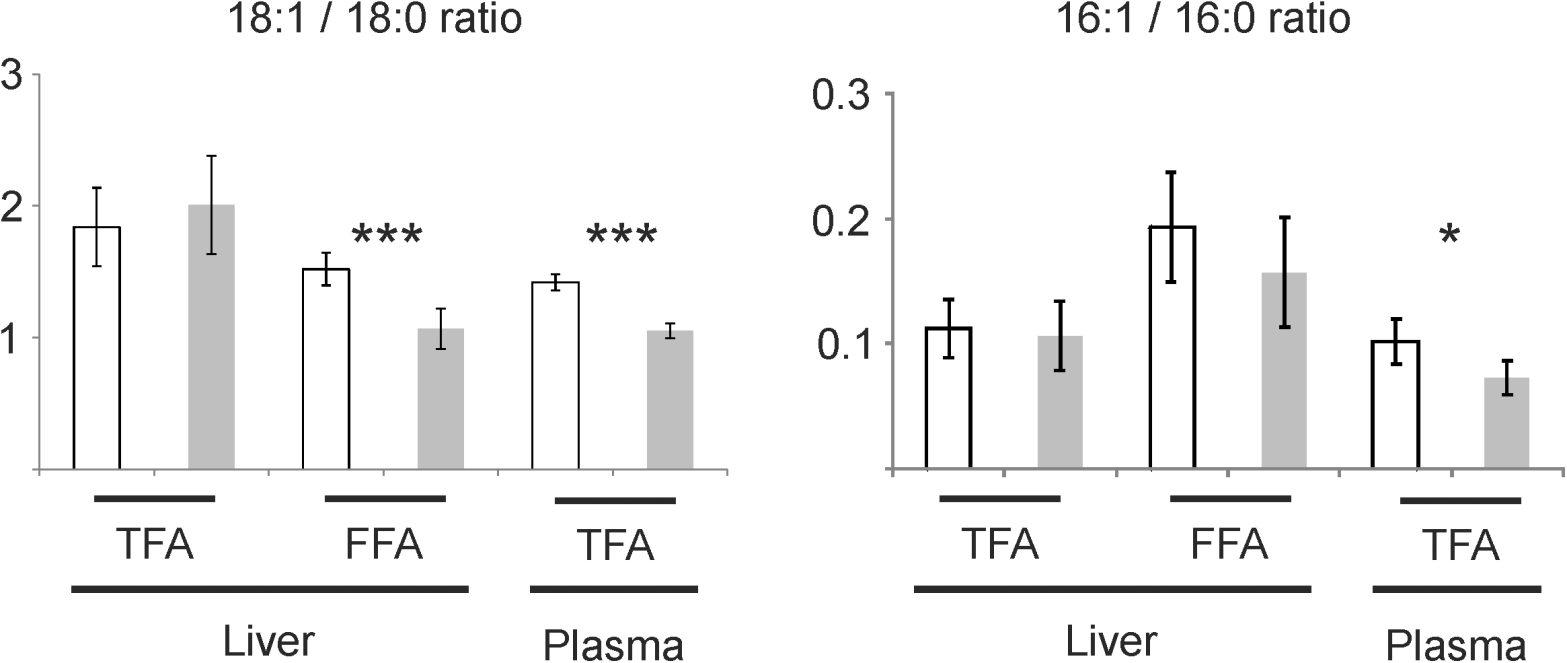
SCD1 related fatty acid ratios in liver and plasma of ATP10D deficient and transgenic mice on high fat diet. Total and non-esterified fatty acids were determined by gas chromatography/mass spectrometry. Mean values are shown together with the standard deviation (n = 5). ATP10D deficient, white bars; ATP10D transgenic, grey bars; TFA, total fatty acids; FFA, free fatty acids. *** p<0.001; *p<0.05.

To substantiate if this regulation of SCD1 is of biological relevance we analyzed hepatic and plasmatic total and FFA composition. Analysis of hepatic and plasma total fatty acids (TFA) revealed a significant relative decrease of palmitate (16:0) and an increase of linoleic acid (18:2 n-6) in TG-mice (Table 3). Furthermore, a significant relative increase of stearate (18:0) and a decrease of oleate (18:1 n-9) was observed in plasma TFA in TG-mice (Table 3). This effect was not apparent in hepatic TFA, however it was found in hepatic FFA. In addition, palmitoleate (16:1 n-7) was always slightly lower in TG-mice (Table 3). The observed increase of stearate (18:0) and decrease of oleate (18:1 n-9) together with the slight decrease of palmitoleate (16:1 n-7) suggests the involvement of SCD1, which forms a cis double bond at the delta-9 position of saturated fatty acyl-CoA substrates like palmitoyl-CoA and stearoyl-CoA. Therefore, we calculated the product/precursor ratio for oleate (18:1 n-9) to stearate (18:0) and palmitoleate (16:1 n-7) to palmitate (16:0), the main delta-9-desaturase reaction products of Scd1. No significant differences were visible when calculating product/precursor ratios of hepatic TFA, but there was a significant decrease in the oleate (18:1 n-9) to stearate (18:0) FFA ratio of TG-mice compared to CTRL-mice (Fig 6). The ratio of palmitoleate (16:1 n-7) to palmitate (16:0) was not significantly different (Fig 6). Interestingly both ratios were significantly elevated in plasma TFA (Fig 6).

**Table 3:** Relative amounts of total and free fatty acids in ATP10D deficient and transgenic mice on high fat diet.

	Hepatic total fatty acids				Hepatic free fatty acids				Plasma total fatty acids			
	CTRL		TG		CTRL		TG		CTRL		TG	
	mean	SD	mean	SD	mean	SD	mean	SD	mean	SD	mean	SD
FA14:0	**0.5%**	0.1%	**0.7%**	0.1%	**1.7%**	0.1%	**2.2%**	0.2%	0.7%	0.1%	0.6%	0.0%
FA16:0	**25.3%**	0.6%	**22.9%**	0.9%	26.8%	1.0%	25.8%	0.7%	**27.5%**	0.8%	**23.4%**	0.7%
FA18:0	8.6%	0.7%	8.7%	1.1%	**11.2%**	0.5%	**14.5%**	1.4%	**7.9%**	0.2%	**10.0%**	0.6%
FA16:1-c9	2.8%	0.6%	2.4%	0.7%	5.2%	1.2%	4.1%	1.1%	**2.8%**	0.5%	**1.7%**	0.3%
FA18:1-c9 (n-9)	15.7%	1.2%	17.1%	1.1%	**17.0%**	0.7%	**15.3%**	0.8%	**11.1%**	0.3%	**10.5%**	0.3%
FA18:2-c9,c12 (n-6)	**24.0%**	1.7%	**27.9%**	1.2%	24.6%	1.7%	25.3%	1.5%	**26.3%**	1.5%	**29.7%**	0.4%
FA20:4-c5,c8,c11,c14 (n-6)	**11.6%**	1.0%	**9.3%**	0.9%	5.0%	0.5%	4.4%	0.5%	15.9%	1.1%	16.2%	0.8%
FA22:6-c4,c7,c10,c13,c16,c19 (n-3)	7.0%	0.6%	6.3%	0.6%	2.9%	0.2%	2.7%	0.4%	**4.4%**	0.2%	**4.8%**	0.1%

Significant differences are shown in bold. CTRL, ATP10D deficient; FA, fatty acid; SD, standard deviation; TG, ATP10D transgenic. n = 5.

**Fig 7:**
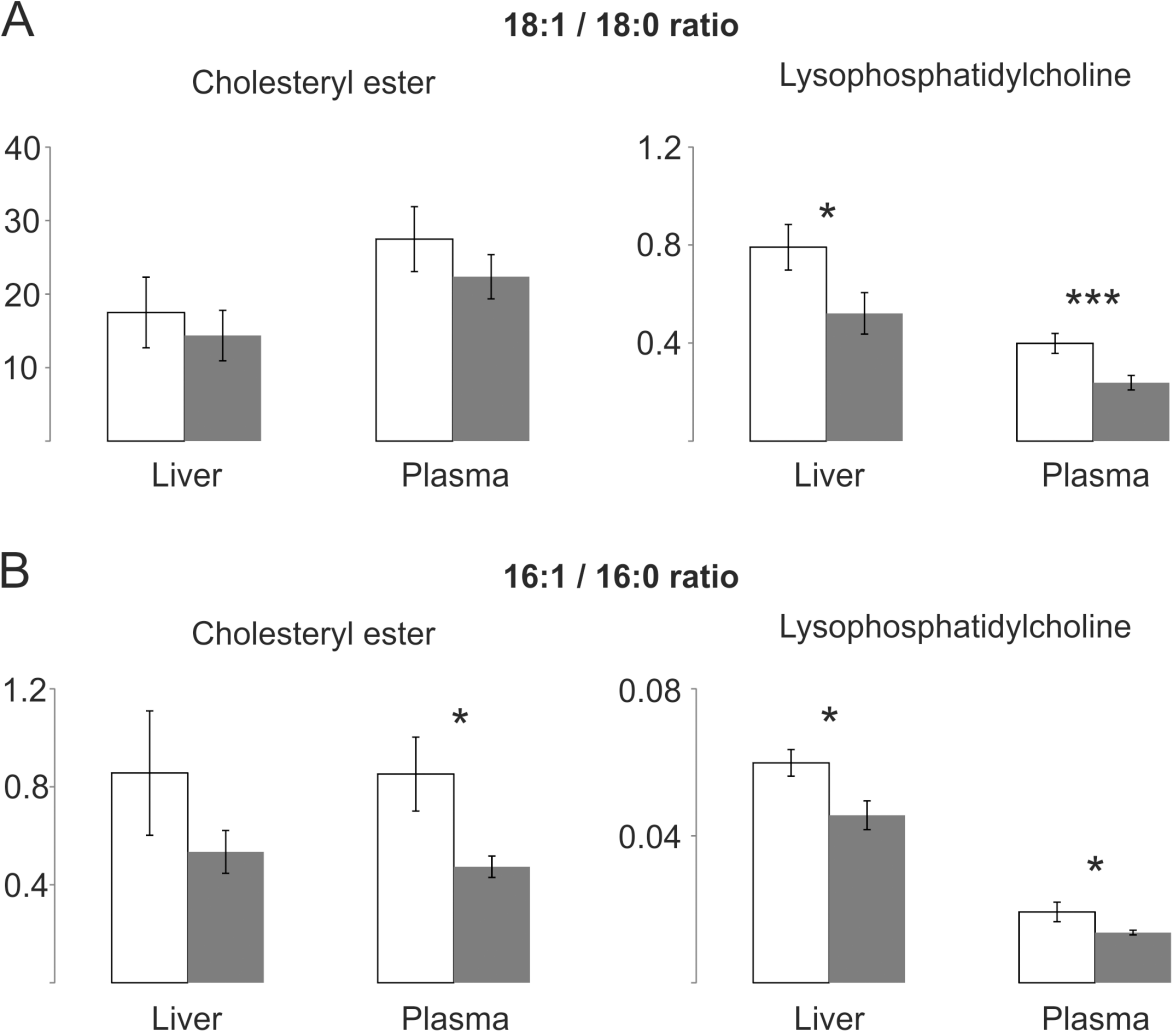
SCD1 related ratios of lipid classes with only one single fatty acid side chain in liver and plasma of ATP10D deficient and transgenic mice on high fat diet. Lipid species were quantified by electrospray ionization tandem mass spectrometry. Mean values are shown together with the standard deviation (n = 4). ATP10D deficient, white bars; ATP10D transgenic, grey bars. *** p<0.001; * p<0.05.

These findings were confirmed in hepatic and plasmatic lipid classes with only one single fatty acid side chain. Similar 18:1 to 18:0 and 16:1 to 16:0 shifts were observed in CE and LPC. While all LPC ratios were significantly different only the shift in plasmatic CE16:1 to 16:0 reached statistical significance (Fig 7).

Overall the 18:1 to 18:0 and 16:1 to 16:0 ratios of CE and LPC in liver and plasma were quite similar to the oleate (18:1 n-9) to stearate (18:0) and palmitoleate (16:1 n-7) to palmitate (16:0) ratios of TFA and FFA, with a tendency towards decreased ratios in TG-mice.

## Discussion

Here we demonstrated that the commonly used C57BL/6J mouse strain, which is deficient for ATP10D, exhibits a less favorable metabolic pattern compared to transgenic mice re-expressing a functional form of *Atp10d*. No significant differences in body weight of ATP10D deficient and transgenic mice were observed when fed a standard chow diet. On a high fat diet TG-mice gain less weight and fat mass and have lower plasma glucose and insulin levels in accordance with better IS. O_2_-consumption/CO_2_-production were found enhanced. In addition they show a distinctly altered lipoprotein pattern, reduced hepatic and plasma HexCer levels, a decreased hepatic FFA to TAG ratio and transcriptional changes of obesity associated genes in liver, including delta-9 desaturase *Scd1*. It has to be noted that the re-expression of ATP10D was conferred by transgenic introduction of a BAC containing the Sv129 *Atp10d*-gene. Analysis of the integration revealed a single copy and we did not observe any noteworthy changes in *Atp10d* expression. Nevertheless, it is possible that the site of integration or other proteins encoded on the BAC might contribute to the observed phenotype.

Interestingly from the tissues analyzed here only the lipid profiles of liver and spleen were altered enough by re-expression of ATP10D to allow clear separation of CTRL-mice and transgenic mice by PCA. Part of these effects could be mediated by the observed reduced SCD1 mRNA and protein expression and the resulting decreased SCD1 product to precursor ratios in ATP10D transgenic mice. The reduced SCD1 expression in transgenic mice is in agreement with previous data demonstrating downregulation of *SCD1* by PUFAs [32,33] and upregulation by insulin, glucose and saturated fatty acids. ATP10D transgenic mice presented a relative gain of linoleic acid (18:2) and loss of palmitate in total hepatic and plasma fatty acids. In contrast to SCD1, *Lpin1* was shown to be suppressed by insulin [34] and in obese insulin resistant mice [35] and we detected increased hepatic *Lpin1* mRNA levels in TG-mice. As it was previously shown that *Scd1* expression is elevated in *Lpin1* null mice [35] the lower *Scd1* expression in TG-mice may also in part be due to increased *Lpin1* expression.

It was also shown that inhibition of SCD1 protects against diet induced obesity, hepatic steatosis and IR [36] and that higher SCD-activity is associated with higher plasma TAG, similar to what we observed in our experiments. Interestingly, upregulation of *Lpin1* reduces hepatic TAG secretion [35]. The finding that TG-mice show lower plasma TAG levels and elevated hepatic TAG may therefore be explained by the observed downregulation of SCD1 and upregulation of *Lpin1*.

The decreased hepatic FFA to TAG ratio indicates improved TAG metabolism due to elevated synthesis and/or reduced degradation and/or secretion in TG-mice. This may lead to decreased release of FFA to the circulation and, therefore to reduced uptake in fat tissue which may explain the observed lesser weight gain in TG-mice. In addition, there may be a link between the observed changes in TAG association to lipoproteins and the decreased plasma TAG levels. It is possible that elevated TAG uptake may prevent modifications of lipoproteins that lead to the formation of LDL-like particles or the prevention of the generation of LDL-like particles may increase TAG uptake.

Previously SNPs in *ATP10D* were shown to be associated with levels of circulating HexCer [4]. Here we could confirm the functional relevance of this finding as TG-mice showed reduced hepatic and plasma HexCer levels compared to CTRL-mice. This may be explained by the observed relative decrease of palmitate (16:0) in TG-mice. Palmitate was shown to increase serine palmitoyltransferase activity and thereby enhance sphingolipid de novo synthesis in a variety of cells (reviewed in [37]). The reduced HexCer levels may contribute to the observed phenotype of TG-mice as different inhibitors of sphingolipid synthesis were shown to improve IR, hepatic steatosis and prevent diabesity in rodent obesity models (reviewed in [2]).

Our findings are in good agreement with reports demonstrating the role of ATP10A (formerly ATP10C), another class 5 P4-type ATPase, in diet-induced obesity, T2D and insulin-stimulated glucose uptake [38–40]. Like ATP10D, ATP10A depends upon interaction with TMEM30A/CDC50A to translocate from the endoplasmatic reticulum to the plasma membrane. There ATP10A revealed PC-specific flipping activity which affects cell shape, adhesion and spreading. However no flippase activity of ATP10D towards PC, phosphatidylserine, PE or SM was detected [41].

In summary, we show here that rescue of ATP10D function in mice on C57BL/6J background leads to reduced HFD induced obesity and IR, altered hepatic expression of lipid-metabolism associated genes, including reduced SCD1 expression and the resulting decreased SCD1 product to precursor ratios, and distinct changes in the plasma lipoprotein pattern.

## Supporting information

S1 Fig Sequencing of Atp10d Exon12 from liver DNA and RNA. (PPTX)

S1 Data Lipid profiling of different tissues. (XLSX)

S1 Table PCA analysis of liver and spleen lipid profiles. (XLSX)

S2 Table MeSH classification of genes with significantly different expression. (XLSX)

## Abbreviations

AGPAT9: 1-acylglycerol-3-phosphate O-acyltransferase 9
ATP10D: ATPase, class V, type 10D
ATP8B1: ATPase, aminophospholipid transporter, class I, type 8B, member 1
BAC: bacterial artificial chromosome
CE: cholesteryl ester
CERS4: ceramide synthase 4
CIDEC: cell death-inducing DFFA-like effector c
FADS: fatty acid desaturase
FFA: free fatty acids
FGF21: fibroblast growth factor 21
HDL: high density lipoprotein
HFD: high-fat diet
HexCER: hexosylceramide
IR: insulin resistance
IS: insulin sensitivity
LDL: low density lipoprotein
LPC: lyso-phosphatidylcholine
LPIN1: phosphatidic acid phosphohydrolase lipin 1
PC: phosphatidylcholine
PCA: principal component analysis
PE: phosphatidylethanolamine
PG: phosphatidylglycerol
SCD1: stearoyl-CoA desaturase 1
SGPP1: sphingosine-1-phosphate phosphatase 1
SM: sphingomyelin
SNP: single-nucleotide polymorphism
SPTLC3: serine palmitoyltransferase, long chain base subunit 3
SREBF1: sterol regulatory element binding transcription factor 1
T2D: type 2 diabetes
TAG: triacylglycerol
TFA: total fatty acids
TMEM30A: transmembrane protein 30A
VLDL: very low density lipoprotein
