# Supplementary material for: Lipidomic and metabolic changes in the P4-type ATPase ATP10D deficient C57BL/6J wild type mice upon rescue of ATP10D function
Source: PLoS One. 2017 May 25;12(5):e0178368. doi: 10.1371/journal.pone.0178368 (PMC5444826; doi:10.1371/journal.pone.0178368)
Supplement: S1 Fig — (PPTX) [file pone.0178368.s001.pptx]

## Slide 1
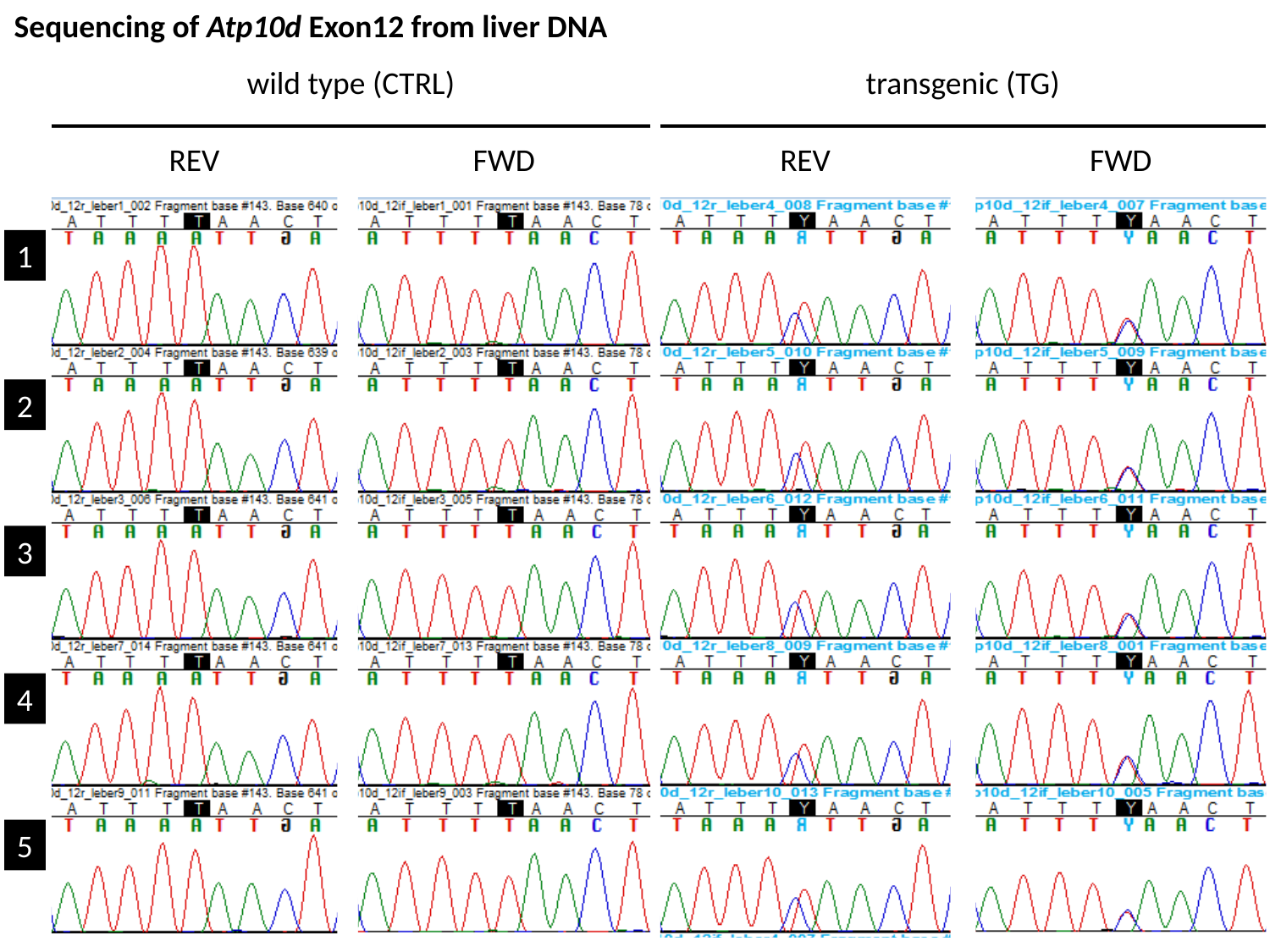

Sequencing of Atp10d Exon12 from liver DNA
wild type (CTRL)
transgenic (TG)
REV
FWD
REV
FWD
1
2
3
4
5

## Slide 2
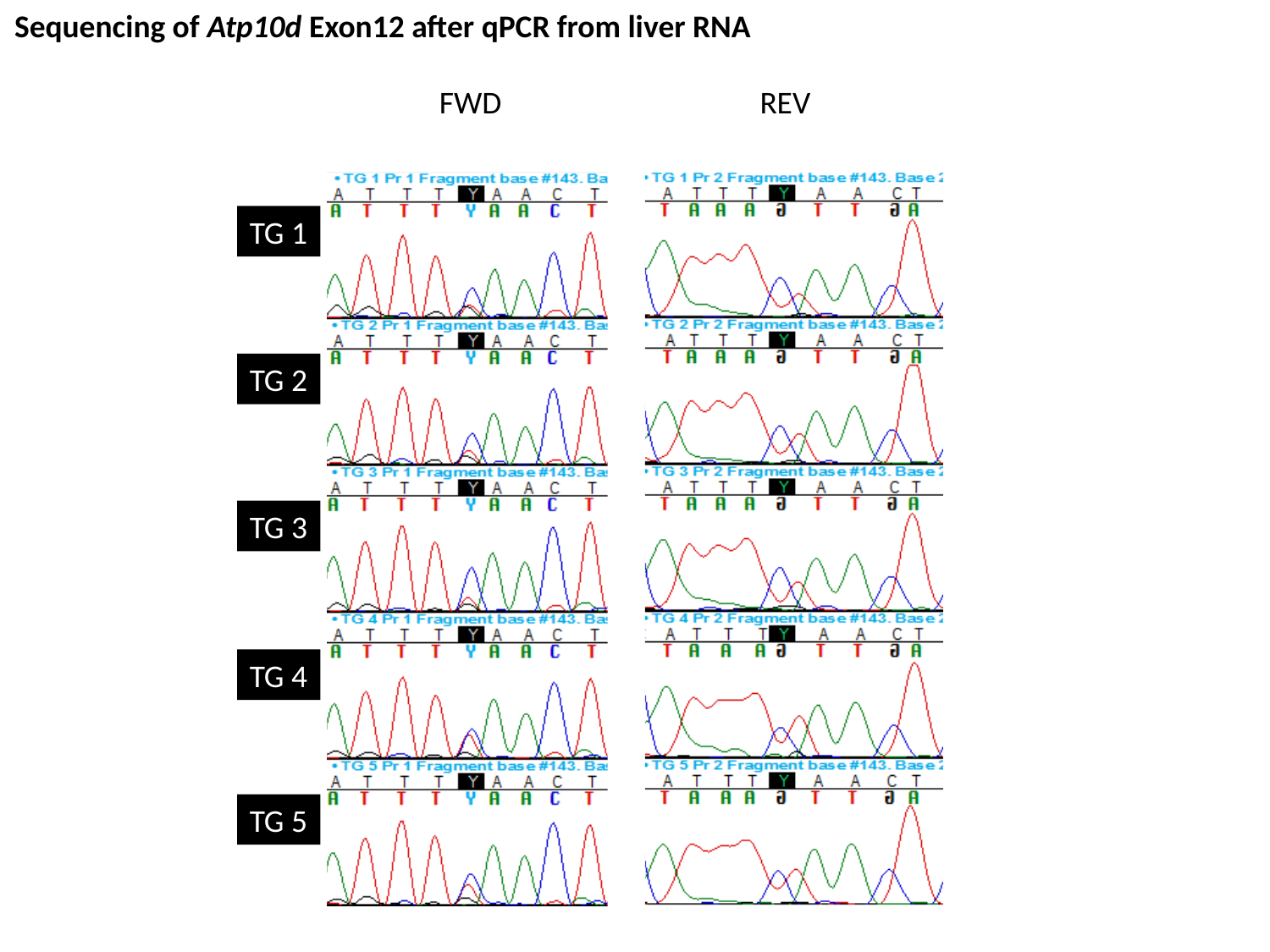

Sequencing of Atp10d Exon12 after qPCR from liver RNA
FWD
REV
TG 1
TG 2
TG 3
TG 4
TG 5
